# Clinical and imaging characteristics of neuroretinitis secondary to cat scratch disease from tertiary centers in Malaysia: a retrospective study

**DOI:** 10.1186/s12348-025-00539-w

**Published:** 2025-11-22

**Authors:** Rajasudha Sawri Rajan, Sangeeta Kuganasan, Nazima Shadaht Ali, Roslin Azni Abdul Aziz, Shelina Oli Mohamed

**Affiliations:** 1 Medical Retina and Uveitis Department, Hospital Shah Alam, Shah Alam, Selangor Malaysia; 2 Ophthalmology Department, Columbia Asia Hospital Seremban, Seremban, Negeri Sembilan Malaysia; 3 Medical Retina and Uveitis Department, Hospital Selayang, Batu Caves, Selangor Malaysia; 4 Department of Ophthalmology, Faculty of Medicine, Universiti Teknologi MARA, Sungai Buloh Campus, Selangor, Sungai Buloh Malaysia

**Keywords:** Neuroretinitis, Cat scratch disease, Bartonella henselae

## Abstract

**Background:**

Cat scratch disease (CSD) is the commonest etiology of neuroretinitis (NR) and is caused by *Bartonella henselae*. The objective of this study was to look at clinical, imaging characteristics and outcomes among serology confirmed Cat scratch disease Neuroretinitis (CSD NR).

**Methods:**

A retrospective review of clinical records of 33 eyes from 28 patients was undertaken over a 5-year period between April 2015 and February 2020.

**Results:**

Mean age at presentation was 34.3 years. Twenty-two (78.6%) had contact with cat. Mean duration of ocular symptoms was 7.0 days. Eighteen (64.3%) had history of fever while 24 (85.7%) complained of blurring of vision. Median baseline logMAR visual acuity was 0.8 (0.2-2.00) and median final logMAR VA was 0.2 (0.00-1.8), *p* < 0.01. Anterior segment inflammation was absent (57.6%) or mild (36.4%). An inflammatory angiomatous optic nerve head lesion was present in 9 (27.3%) eyes. Seventeen (51.5%) eyes had retinal infiltrates or focal retino-choroiditis. This was seen in 66.7% eyes presenting within a week and was absent in 77.8% presenting after a week (*p* = 0.034). Eight (24.2%) eyes had no macula exudates at presentation out of which 7 had peripapillary subretinal fluid (SRF) on optical coherence tomography (OCT). Median baseline oct central subfoveal thickness (CST) was 303 μm (213–1159) and median final CST was 262 μm (193–344), *p* < 0.001. Hyperreflective vitreous dots on OCT were present in 16 (50.0%) with no clinical evidence of vitritis or vitreous cells in 18.75%. Peripapillary SRF was seen in 26 (81.3%), subfoveal SRF in 23 (71.9%), macula intraretinal fluid (IRF) in 12 (37.5%) and abnormal foveal contour in 22 (68.8%). Fluorescein angiography showed disc leakage in 10 out of 15 eyes (66.7%). All patients received treatment, 17(60.7%) with oral antibiotics and 11 (39.3%) with additional oral steroids.

**Conclusion:**

Presenting visual acuity was moderate to poor with good final visual outcomes. Severe anterior uveitis was uncommon. Absence of baseline macular exudates maybe associated with peripapillary subretinal fluid. Retinal infiltrates or focal retino-choroiditis were common but seen early in presentation. Hyperreflective dots in the vitreous on OCT may precede clinical visualization of vitreous involvement. Significant reduction in CST on OCT was seen at six weeks.

## Introduction

Neuroretinitis is characterized by unilateral optic disc oedema with macula exudates in a star pattern. It was mislabeled as ‘stellate maculopathy’ before subsequent studies showed that the origin of the inflammation and exudation was primarily the optic disc and not the macula [1]. Furthermore, the macula star was found to not always be present during the initial visit. The classic macula star may present at later times during the disease course, and it is usually in a radial configuration [2]. 

There are a variety of predominantly infectious and less commonly non-infectious causes that can lead to the development of neuroretinitis [2]. However, the most common cause is cat scratch disease (CSD) comprising up to almost 90% of all neuroretinitis cases [3–5]. CSD was first described in 1950 by Debre et al.^6^ This disease is usually self-limiting, systemic in nature caused by the organism Bartonella Henselae (B. henselae). It is the most common variant, which is a fastidious, gram-negative intracellular bacillus [7, 8]. Diagnosis of CSD NR is usually made from clinical findings and supported with serological detection of B.henselae Immunoglobulin M (IgM) and/or Immunoglobulin G (IgG) [9, 10, 24] Limitations of this serological testing is its low sensitivity. Furthermore, it is time sensitive whereby IgM is detectable only in 50% at 3 months and IgG is only detectable in 25% of patients at one year. IgG has a high specificity but variable sensitivity and is regarded as the gold standard.

Although most patients with CSD NR demonstrate complete resolution with a favourable visual prognosis, there are occasionally those who have persistent poor visual recovery [3]. 

The primary aim of this study was to look at the clinical, imaging characteristics, disease pattern and outcomes among patients with CSD NR with a confirmed serology from our centers. It is hoped that the findings would fill in gaps of our understanding towards this increasingly common entity.

## Methodology

This was a retrospective, observational study looking at the case records and images of all patients who were diagnosed with CSD NR over a 5-year period from April 2015 to February 2020. The two tertiary referral hospitals for uveitis involved in this study were Hospital Shah Alam and Hospital Selayang.

The study was conducted following the tenets outlined in Declaration of Helsinki and was registered in the National Medical Research Register NMRR ID-24–02740−7WK.

Patients were diagnosed to have CSD NR if they fulfilled the classical triad of reduced vision associated with optic disc edema and macula star at presentation or during the course of the disease. A positive history of physical contact with cat was supportive of the diagnosis but not mandatory. This could include scratches, bites or licks from the cats. The patients were confirmed to have CSD NR if the clinical findings were supported with a positive Bartonella serology. Other causes of neuroretinitis were excluded such as ocular Tuberculosis, Neurosyphilis, ocular Toxoplasmosis, DUSN and non-infectious causes like ocular sarcoidosis, malignant hypertension, and less commonly posterior scleritis. The Bartonella serology conducted in these centers are the Indirect Fluorescent Antibody (IFA) test for both IgG and IgM. This is considered a reliable method with specificity reaching 95%.^9^ Bartonella serology was considered significant if the IgM titre was more than 1:24, and IgG titre more than 1:128 [10]. 

The inclusion criteria were patients who had a diagnosis of CSD NR at the end of their follow up with available serology results. These patients should have had at least 6 weeks of follow up from time of presentation. Final clinical outcomes were recorded at the 6 weeks follow-up from baseline. Patients who had incomplete documentation of their ocular findings, Optical Coherence Tomography (OCT) imaging, unavailable Bartonella test results or non-compliance to treatment or follow up were excluded.

The information collected included age, gender, ethnicity, physical contact with felines, and any systemic symptoms such as fever or lymph node swelling. The best corrected visual acuity documentation would include both at presentation, and at the end of follow up. Slit lamp examination to assess features of co-existing anterior segment inflammation such as keratic precipitates, anterior chamber cells, flare or hypopyon and posterior synechiae were documented. Severe anterior uveitis was defined as presence of hypopyon, flare, anterior cells of more than 2 + based on SUN grading and posterior synechiae. If they had other ancillary testing done such as Fundus Fluorescein Angiography (FFA) or Colour Fundus Photography (CFP), these were documented as well.

Data analysis was performed using SPSS version 29.0. Chi-square test/Fisher’s exact test was used for the comparison of categorical variables and two-tailed Student’s t-test/Wilcoxon signed-ranks test for comparison of continuous variables.

## Results

There were 33 eyes from 28 patients with a serology confirmed diagnosis of CSD NR. The baseline demographics and clinical features are shown below (Table 1). The youngest was 19 years while the oldest was 65 years. Majority of patients were of the Malay race (92.9%) while only one patient each was Chinese (3.6%), and Indian (3.6%).

**Table 1:** Baseline dermographic and clinical features in patients with CSD neuroretinitis

	Patients (*n* = 28)		
	Mean (SD)	Median (IQR)	n (%)
Age, years (mean ± SD)	34.3 (12.47)		
Less than 40 years			20 (71.4)
Male			20 (71.4)
Malay race			26 (92.9)
Student			5 (17.9)
Working			20 (71.4)
Scratched by cat			11 (39.3)
Duration Ocular Symptoms, days		7.7.0 (4.0, 14.0)	
Duration Of Ocular Symptoms			
*≤* 1 week			16 (57.1)
>1 week			12 (42.9)
History Of Fever			18 (64.3)
Blurring Vision			24 (85.7)
Unilateral			23 (82.1)
Bartonella IgM > 1:24			23 (82.1)
Bartonella IgG > 1:128			24 (85.7)
Treatment			
Antibiotics only			17 (60.7)
Antibiotics + systemic steroids			11 (39.3)
Choice of antibiotics			
Doxycycline			18 (64.3)
Others			10 (35.7)

Among twenty-two (78.6%) patients who gave a history of contact with cat or other animals, only 11 (39.3%) admitted to being scratched, bitten or licked. Median duration of ocular symptoms was 7.0 days (4 to 14 days) and 16 (57.1%) presented within one week or less. 18 (64.3%) patients gave a history of fever within three months of presentation. Twenty-four (85.7%) complained of blurring of vision. Neuroretinitis was unilateral in 23 (82.1%). Left eye was affected in 14 (42.4%) and right eye in 9 (27.3%).

All 28 patients had a positive Bartonella serology. Out of this, 19 (67.9%) had both IgG and IgM positivity, 5 (17.9%) had only IgG positivity and 4 (14.3%) had only IgM positivity.

Patients who had a history of fever within the preceding three months were compared with those who did not. A history of fever was significantly associated with contact with cats (*p* = 0.03) and a positive Bartonella IgM serology titre of > 1:24 (*p* = 0.03).

All patients received treatment. Seventeen (60.7%) were treated with systemic antibiotics and 11 (39.3%) required additional oral steroids when optic nerve dysfunction was present or if response was slow with antibiotics (Table 1). One patient received intravenous steroids. Oral Doxycycline monotherapy at 100 mg twice daily for at least 6 weeks was used in 18 (64.3%) while the remaining patients received additional Rifampicin for severe cases or other choices of antibiotics such as Trimethoprim/Sulfamethoxazole when Doxycycline was not tolerated.

### Clinical findings

Median baseline Logarithm of the Minimum Angle of Resolution (LogMAR) visual acuity (VA) was 0.8 (0.2–2.00.2.00) and median final logMAR VA was 0.2 (0.00–1.8.00.8), *p* < 0.01. The presenting and final visual acuity were stratified into three categories of Snellen visual acuity (Table 2). At baseline 23 eyes (69.7%) presented with moderate to poor VA (6/15 to < 6/60) and this reduced to 11 eyes (33.3%) at the outcome. The proportion of patients with good presenting VA (6/6 to 6/12) increased from 10 (30.3%) to 22 eyes (66.7%) at final follow-up.

**Table 2:** Baseline and final Snellen visual acuity stratified in three groups

Visual acuity	Good (6/6–6/12) *n*(%)	Moderate (6/15 − 6/60) *n*(%)	Poor (< 6/60) *n*(%)
Presentation	10(30.3%)	16(48.5%)	7(21.2%)
Outcome (6 weeks)	22(66.7%)	10(30.3%)	1(3.0%)

The clinical characteristics of eyes with CSD NR were summarized (Table 3). Results for relative afferent pupillary defect (RAPD) at presentation was available for 24 eyes and was positive in 14 (42.4%). Baseline colour vision was available for 27 eyes out of which 19 (57.6%) were abnormal. Signs of severe anterior uveitis were less common. Keratic precipitates were absent in 26 (78.8%) eyes while fine non-granulomatous keratic precipitates were seen in the remaining 7 (21.2%). Anterior chamber reaction was generally absent or mild. Based on SUN grading [29], grade 0 was seen in 19 (57.6%) eyes, 1 + in 12 (36.4%) and 2 + in the remaining 2(6.1%) eyes. Posterior synechiae was not seen in all 33 (100%) eyes. Anterior vitreous cells were present in 18(54.5%) eyes. Twenty-three (69.7%) eyes had no vitritis while, 4(12.1%) eyes each had grade 0.5 + and grade 1 + vitritis respectively. The remaining 2 (6.1%) eyes had grade 2 + vitritis.

**Table 3:** Clinical characteristics of eyes with CSD neuroretinitis

	Eyes (*n* = 33)		
	Median (min-max)	n	(%) p
Bilateral Eyes		10	(30.3)
Baseline LogMAR VA	0.8 (0.20–2.00)		
Final LogMAR VA	0.2 (0.00–1.80)		<0.01
∗RAPD		14	(42.4)
*δ* Colour vision abnormal		19	(57.6)
Absent keratic precipitates		26	(78.8)
*AC_Reaction*:			
nil		19	(57.6)
1+		12	(36.4)
Nil posterior_synechiae		33	(100.0)
Vitreous_cells present		18	(54.5)
Vitreous_haze:			
Gd 0		23	(69.7)
0.5 + to 1+		8	(24.2)
Retinal infiltrate/focal retino-choroiditis/		17	(51.5)
Retinal vasculitis		5	(15.2)
Venous_dilatation		7	(21.2)
OD angiomatous lesion		9	(27.3)
Diffuse OD swelling		32	(97.0)
*Macula_at_presentation*:			
Complete star		15	(45.5)
Partial star		10	(30.3)
No star		8	(24.2)
Topical_steroids		15	(45.5)

*OD* Optic disc, *RAPD* Relative afferent pupillary defect

∗RAPD findings were not available for 9 eyes (*n* = 24)

***δ*** Colour vision results were not available for 6 eyes (*n* = 27)

**Fig. 1:**
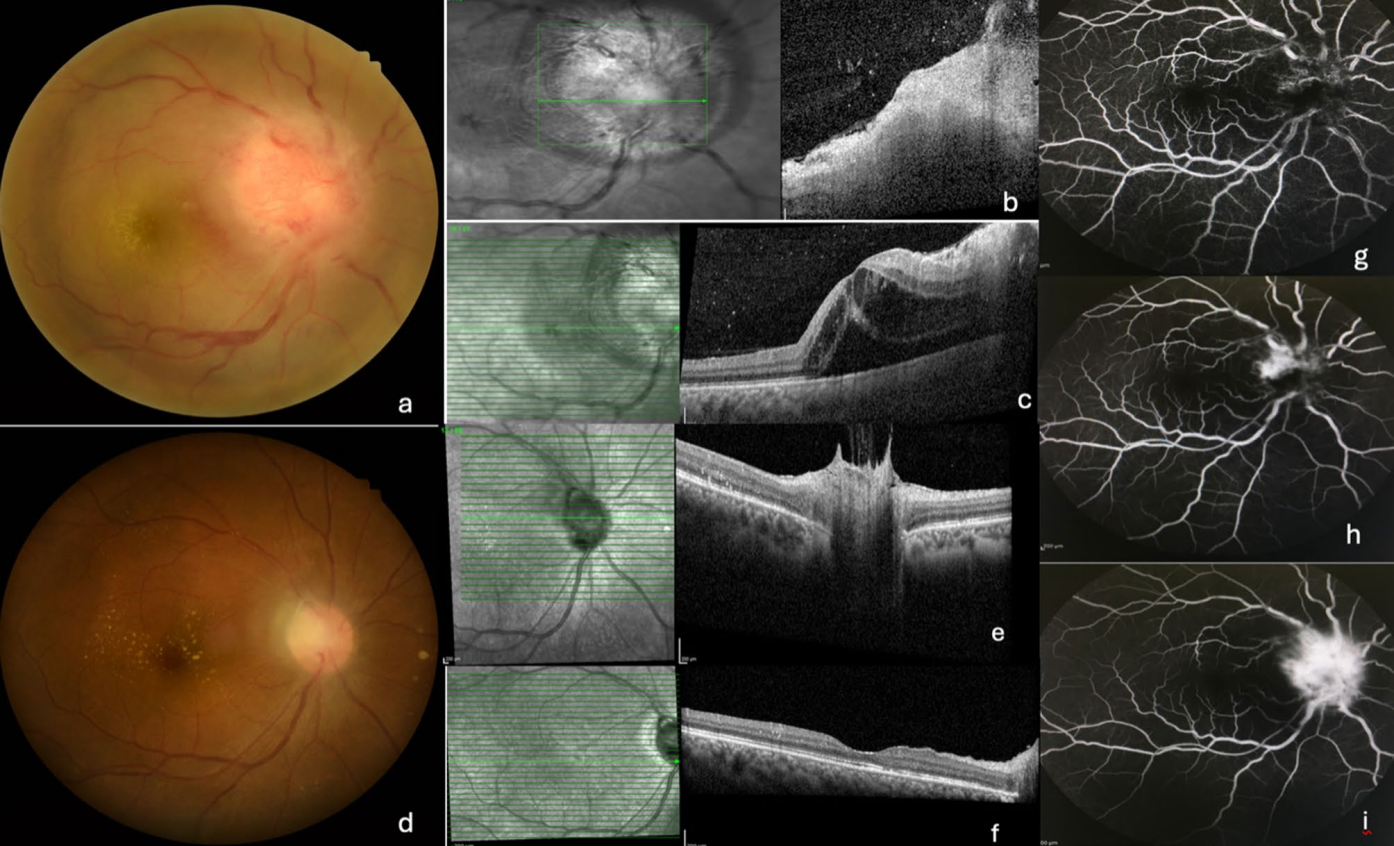
Fundus Photo, OCT and FFA of a patient with CSD neuroretinitis. A 43 year old female with CSD NR. **a** baseline VA 6/60. Fundus photo showing large inflammatory angiomatous lesion over optic disc, partial macula star, retinal haemorrhages along inferotemporal vein. **b** baseline OCT optic nerve head showing increased thickness, increased hyperreflectivity with vitreous dots. **c** baseline macula OCT showing vitreous dots, macula IRF and SRF extending from peripapillary to subfoveal region. **g-i** FFA showing early fluorescence of abnormal vessels over optic nerve head with progressive leakage at later phases. **d** VA improved to 6/9 at 12 weeks with resolution of angiomatous lesion and resolving macula exudates. **e** OCT showing resolution of thickness and hyperreflectivity with overlying areas of vitreous traction (**f**) OCT showing complete resolution of IRF and SRF with foveal thinning

On fundus examination, 32 (97.0%) eyes had generalized optic disc swelling while one (3.0%) had sectoral swelling. An angiomatous lesion over the optic disc was documented in 9 (27.3%) eyes. (Fig. 1). Retinal infiltrates or focal retino-choroiditis were present in 17 (51.5%) eyes, vasculitis in 5 (15.2%) and venous dilatation in 7(21.2%). Macula exudates were classified based on presence of a complete star, incomplete star or absent at presentation. Macula star was present in 25 (75.8%) out of which 15 (45.5%) showed a complete star and in 10 eyes (30.3%) it was incomplete. Eight (24.2%) eyes had no macula exudates at presentation but developed it along the course of the disease. Out of this, 7 eyes had peripapillary subretinal fluid (SRF) and 5 had macula SRF on OCT at presentation.

Baseline OCT findings were available for 32 eyes (Table 4). Hyperreflective dots were present in the vitreous in 16 (50.0%). Out of this, 6 eyes (18.75%) had no evidence of anterior vitreous cells or vitreous haze on examination. SRF was present at the peripapillary region in 26 (81.3%) and at subfoveal region in 23 (71.9%). Intraretinal fluid at the macula was present in 12(37.5%) eyes all of which had presence of subretinal fluid at both the peripapillary and subfoveal region. An abnormal foveal contour was present in 22 (68.8%) eyes.

**Table 4:** Optical coherence tomography (OCT) and fundus fluorescein angiography (FFA) features in CSD neuroretinitis

		Eyes		
	Median(Min-Max)	n	(%)	p
OCT		**32**		
OCT BASELINE_CST	303.0(213–1159)um			
OCT FINAL CST	262.0 (*193–344*)um			<0.01
Juxtapapillary_subretinal fluid				
Present		26	(81.3)	
Absent		6	(18.7)	
Subretinal_fluid_macula_				
Present		23	(71.9)	
Absent		9	(28.1)	
Hyperreflective_dots_vitreous				
Present		16	(50.0)	
Absent		16	(50.0)	
Intraretinal fluid macula				
Present		12	(37.5)	
Absent		20	(62.5)	
Foveal contour				
Normal		10	(31.2)	
Abnormal		22	(68.8)	
FFA		**15**		
Capillary Non-perfusion				
Present		1	(3.0)	
Absent		14	(93.3)	
Vasculitis				
Present		10	(66.7)	
Absent		5	(33.3)	
Cystoid macula edema				
Absent		12	(80.0)	
Not available		3	(20.0)	
FFA: _Hot_disc_				
Present		9	(60.0)	
Absent		1	(36.7)	

The median baseline central subfoveal thickness (CST) was 303 μm (213–1159) and the median final CST was 262 (193–344), *p* < 0.001.

Twenty-one eyes (65.6%) had a CST between 250 and 500 μm at baseline and 6 (18.8%) had a CST of more than 500 μm. CST was less than 250 μm in 5 (15.6%) eyes at baseline and this number increased to 10 (32.3%) after 6 weeks. 7 out of 10 of these eyes had baseline subfoveal fluid.

**Fig. 2:**
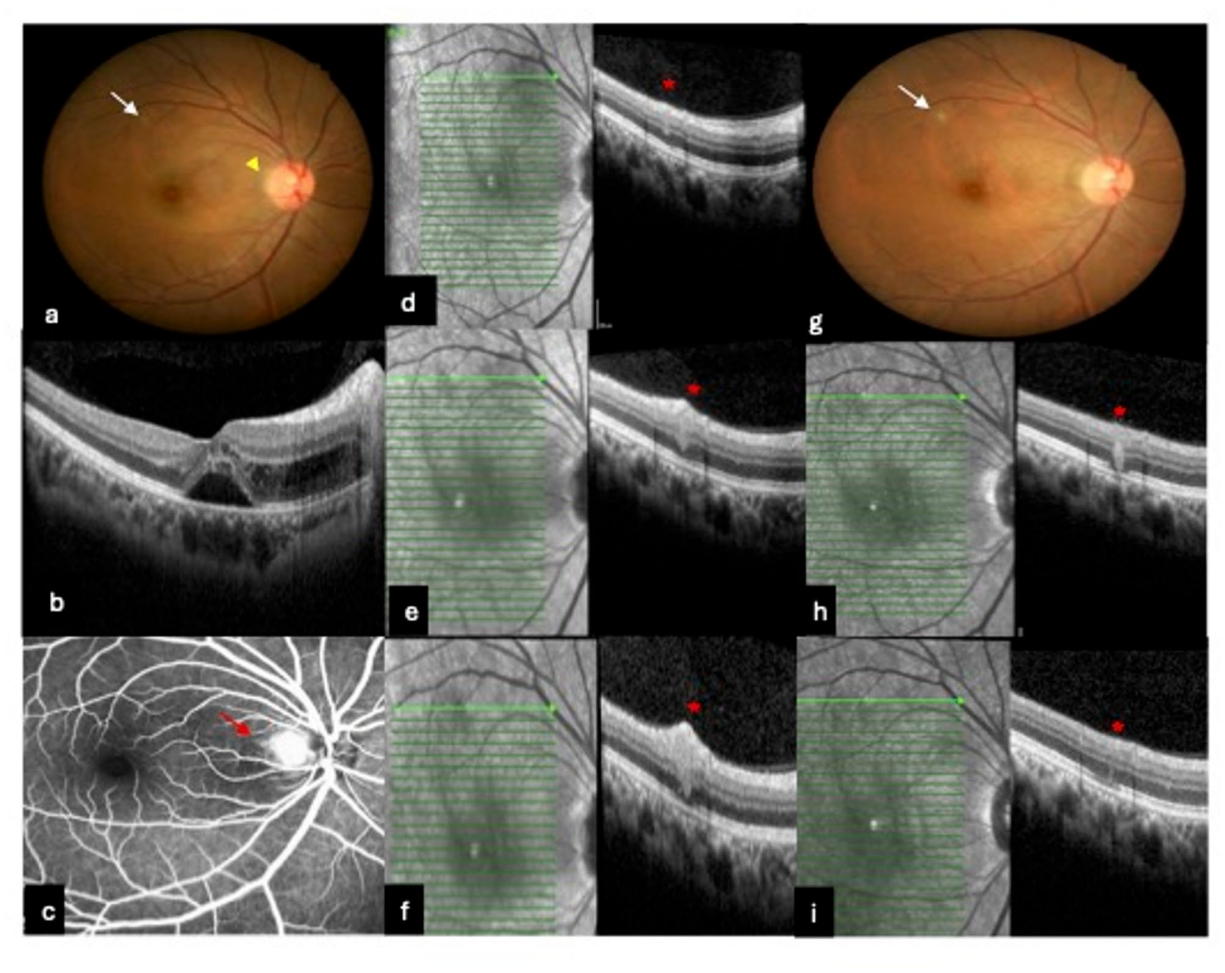
Early presentation of CSD NR and OCT across superficial retinal infiltrate. A 39 year old lady with early presentation of CSD NR within 2 days (**a**) Hyperaemic optic disc with vague overlying angiomatous lesion temporally (yellow arrowhead). Superficial retinal infiltrate superotemporally (white arrow). Note absence of macula exudates. **b** OCT at baseline showing macula SRF with IRF at ONL extending from peripapillary to parafoveal region (**c**) FFA showing hyperfluorescence and leakage of angiomatous lesion (red arrow). d & e) OCT across the superficial retinal infiltrate (red star): at baseline (**d**) fine streak of hyperreflectivity from RNFL to ONL and day 7 (**e**): more prominent hyperreflective lesion. **f** OCT day 9: hyperreflectivity extending downward obscuring ellipsoid layer (EL) **g** Fundus photo at Day 7: Presence of stellate macula exudates nasal to fovea and more prominent superficial retinal infiltrate. **h** OCT Day 21 showing more confined hyperreflective lesion involving inner plexiform to EL. **i** Day 55: thin line of hyperreflectivity with focal loss of EL

OCT characteristics of retinal infiltrates or focal retino-choroiditis were not analyzed as relevant images were not available for many cases. However, OCT sections across these lesions from few available cases showed a hyperreflective lesion extending from the retinal nerve fiber layer (RNFL) to the ellipsoid layer (EL) which varied in size and depth of involvement with duration from onset. (Fig. 2).

Fundus fluorescein angiography results were available for 15 (45.5%) eyes (Table 4). Out of this, 10 (66.7%) eyes had leakage from the optic disc and retinal vasculitis. Cystoid macula edema was not seen in all eyes.

Five patients with 10 eyes had bilateral CSD NR. All had history of contact with cat and presented within one week from onset of symptoms. Four had a history of fever. All 5 had raised erythrocyte sedimentation rate (ESR) levels for their age ranging between 48 and 86 mm/hour and at least a Bartonella IgM positivity. Retinal infiltrates or focal retino-choroiditis were present in all these eyes. Baseline OCT showed peripapillary SRF in all eyes, subfoveal SRF in 8 eyes and presence of macula IRF in 4 eyes. Four eyes required oral steroids in addition to antibiotics.

### Inflammatory angiomatous optic nerve head lesion

The clinical, OCT features and treatment pattern were compared between eyes that were found to have an angiomatous lesion over the optic nerve at baseline and eyes without the lesion (Table 5). Presence of this optic nerve head lesion at baseline was significantly associated with the use of systemic steroids (*p* = 0.05), abnormal colour vision at presentation (*p* = 0.008), abnormal foveal contour (*p* = 0.02) on OCT, macula subretinal fluid (*p* = 0.044) but not peripapillary subretinal fluid (*p* = 0.19) or macula intraretinal fluid (*p* = 0.16). However, further analysis with multiple logistic regression was not possible due to small sample size.

**Table 5:** Clinical characteristics and association between presence of OD angiomatous lesion

	OD angiomatous lesion		
	Present *n* = 9	Absent *n* = 24	p-value
		n (%)	n (%)
Baseline visual acuity			
6/6–6/12	1 (11.1)	9 (37.5)	0.147^b^
< 6/12	8 (88.9)	15 (62.5)	
RAPD			
Yes	5 (55.6)	9 (37.5)	1.000^b^
No	3 (33.3)	7 (29.2)	
Colour vision			
Normal	0 (0.0)	8 (33.3)	0.008^b^
Abnormal	9 (100.0)	10(41.7)	
Not available	0 (0.0)	6 (25.0)	
Macula exudates			
Complete star	2 (22.2)	13(54.2)	0.247^b^
Partial star	4 (44.4)	6 (25.0)	
No star	3 (33.3)	5 (20.8)	
OCT juxtapapillary SRF			
Yes	9 (100.0)	17(70.8)	0.190^b^
No	0 (0.0)	6 (25.0)	
Not available	0 (0.0)	1 (4.2)	
OCT subretinal fluid macula			
Yes	9 (100.0)	14(58.3)	0.044^b^
No	0 (0.0)	9 (37.5)	
Not available	0 (0.0)	1 (4.2)	
OCT dots in vitreous			
Yes	7 (77.8)	9 (37.5)	0.142^b^
No	2 (22.2)	14(58.3)	
Not available	0 (0.0)	1 (4.2)	
OCT macula intraretinal fluid			
Yes	5 (55.6)	7 (29.2)	0.160^a^
No	4 (44.4)	17(70.8)	
OCT foveal contour			
Normal	0 (0.0)	10(41.7)	0.020^a^
Abnormal	9 (100.0)	14(58.3)	
ORAL STEROIDS			
Yes	6 (66.7)	7 (29.2)	0.050^a^
No	3 (33.3)	17(70.8)	

^a^Chi-square

^b^Fisher’s exact

### Association between timing of presentation with clinical and imaging features

We explored the association between timing of presentation with clinical and imaging features (Table 6). There was a significant association between timing of presentation with presence of retinal infiltrates or focal retino-choroiditis (*p* = 0.034) and baseline macula intraretinal fluid on OCT (*p* = 0.017) but not peripapillary SRF (*p* = 0.307) or macula SRF (*p* = 0.663). Ten out of fifteen eyes that presented within a week following onset of symptoms had retinal infiltrates or focal retino-choroiditis while 14 out of 18 eyes that presented later than a week did not have these lesions. Baseline macula intraretinal fluid was seen in 9 out of 15 eyes that presented early within one week while 15 out of 18 eyes that presented after a week did not have IRF.

**Table 6:** Association between timing of presentation with retinal and OCT features

	TIMING OF_PRESENTATION_FROM OCULAR SYMPTOMS			
	< 1 week	1–2 weeks	>2 weeks	*p*-value
	*n* (%)	*n* (%)	*n* (%)	
RETINAL INFILTRATES/FOCAL RETINO-CHOROIDITIS				
Present	10 (66.7)	4 (26.7)	0 (0.0)	0.034^b^
Absent	5 (33.3)	11 (73.3)	3 (100.0)	
MACULA EXUDATES				
Complete star	6 (40.0)	8 (53.3)	1 (33.3)	0.693^b^
Incomplete star	4 (26.7)	4 (26.7)	2 (66.7)	
Absent	5 (33.3)	3 (20.0)	0 (0.0)	
OCT PERIPAPILLARY SRF				
Yes	14 (93.3)	10 (66.7)	2 (66.7)	0.307^b^
No	1 (6.7)	4 (26.7)	1 (33.3)	
Missing	0 (0.0)	1 (6.7)	0 (0.0)	
OCT BASELINE MACULA SRF				
Present	12(80.0)	9 (60.0)	2 (66.7)	0.663^b^
Absent	3 (20.0)	5 (40.0)	1 (33.3)	
Missing	0 (0.0)	1 (6.7)	0 (0.0)	
OCT_BASELINE_MACULA_IRF_				
Yes	9 (60.0)	2 (13.3)	1 (33.3)	0.017^b^
No	6 (40.0)	13 (86.7)	2 (66.7)	

^b^Fisher’s exact test

## Discussion

*Bartonella henselae* is a ubiquitous, fastidious gram-negative bacteria with world-wide distribution transmitted predominantly by the cat flea, *Ctenocephalides felis*. The primary reservoirs are domestic cats which transmit the infection to humans through bites, licks or scratches resulting in cat scratch disease (CSD). Ocular manifestation develops in 5 to 10% and posterior segment findings are common [15]. Neuroretinitis has been reported in 1 to 2% of patients with CSD [5, 8]. While the exact pathogenesis is still unclear, optic nerve involvement may be a result of direct infection by Bartonella, an immune-mediated response to the infection or a combination of both [14]. 

CSD is the commonest etiology of neuroretinitis in immunocompetent individuals. This study described the clinical characteristics, ocular imaging features and outcomes in a series of immunocompetent adults with CSD neuroretinitis.

### Demographics and clinical presentation

Males comprised almost three quarters of the patients. Male predominance of CSD has been reported between 58 and 60% from a US national database comprising more than 2000 patients. (15) Smaller series have reported almost equal gender distribution with slight predilection towards males (3). Consistent with another series from Malaysia (13), Malays were the predominant race in all except two patients from this multi-ethnic series. This is explained by felines being the preferred domestic pet among Malays due to religious reasons and stray cats being quite common in Malaysia [25]. 

In our series with serology proven CSD Neuroretinitis, 6 out of 28 (21.4%) denied history of contact with cats. Hande C et al.^12^ described three cases of serology proven neuroretinitis with no prior contact with cats or other animals, which resolved with no recurrence. Likewise, exposure to cats or animals in ocular CSD has been reported in only 67% to 83% of cases [3, 17]. This is supported by potential direct transmission to humans via tick bites [15]. Hence, *B. henselae* infection should still be considered in patients without prior exposure to felines or other animals. We recommend that the term *CSD Neuroretinitis* should be revised to *Bartonella NR* to include patients with no specific prior cat exposure including scratches or bites.

More than half presented within one week from onset of symptoms with blurring of vision consistent with other studies [8, 22]. Fever was reported in almost two thirds within the preceding 3 months like other reports, which underscores the importance of eliciting this history in patients who usually seem well [13, 19]. Prolonged fever in CSD may be a sign of disseminated infection with other systemic symptoms like myalgia, arthralgia, malaise and weight loss [16]. A history of fever was more likely to be reported in patients with recent infection confirmed by positive Bartonella IgM serology and positive contact with cats in our series. Although lymphadenitis is the most common systemic manifestation of CSD, data for this was scarce and unavailable from our retrospective series [8]. 

Like other reports, unilateral presentation was predominant in 82% of patients [3, 14]. Although less common, bilateral neuroretinitis has been reported in both the immunocompetent and immunocompromised [8, 20]. However, this should also prompt further investigation to rule out masqueraders such as malignant hypertension, raised intracranial pressure or Idiopathic intracranial hypertension [18]. 

### Clinical features

Consistent with other studies, presenting vision was moderate to poor with good final outcomes in the majority [13, 17, 20]. One patient in this series had a final VA of less than 6/60 (Table 2). This was attributed to a delay in presentation 12 days after the onset of symptoms with poor presenting VA and optic nerve dysfunction characterized by a positive RAPD and abnormal colour vision. In immunocompetent patients CSD is known to be self-limiting with excellent visual prognosis [19]. Poor visual outcomes maybe attributed to optic nerve dysfunction or macula atrophy [14]. A positive RAPD with abnormal colour vision at the onset and during follow-up would facilitate this differentiation.

Anterior segment inflammation was mild or absent. Similar findings have been reported by Ksiaa et al.^8^ Fine keratic precipitates were present in only 1 in 5 eyes. Likewise, anterior chamber reaction was absent in more than half while none of the eyes had posterior synechiae. The presence of severe signs of anterior uveitis in an adult eye with Neuroretinitis could prompt an etiology other than *Bartonella henselae*.

An inflammatory angiomatous lesion over the optic nerve head was present in 27% of eyes in our study. This is lower than reported by Zohar who found 40% of eyes with this lesion [3]. It is possible that this finding may have been under-reported in our retrospective series. The unique quality of *Bartonella henselae* is its ability to cause vasoproliferative lesions, a pathological angiogenesis resulting in the formation of new capillaries from pre-existing ones [21]. Since the optic nerve head is the predominant site of inflammation in neuroretinitis, this may explain the formation of these lesions [20]. We found significant association in eyes with these angiomatous lesions and abnormal colour vision, abnormal foveal contour, presence of macula SRF but not peripapillary SRF and use of systemic steroids. This is compatible with the tendency of these lesions to leak profoundly resulting in inflammation at and around the optic disc. The resultant fluid flows directly into the outer plexiform space, passing through the external limiting membrane to accumulate beneath the neurosensory retina subfoveally.

Macular exudates were not seen in 8(24%) eyes at baseline in our study while optic disc swelling was present in all. However, peripapillary SRF on OCT was present in 7 of these eyes and macula SRF in 5. It is postulated that inflammation of the optic disc and the vasculature surrounding it leads to fluid exudation in the peripapillary retina resulting in serous detachment with subsequent appearance of macular exudates in a partial or complete star pattern around the fovea [8]. This highlights the importance of performing OCT around the optic disc and not just the macula in eyes with optic disc swelling where the presence of fluid could precede onset of clinically apparent macula exudates.

Retinal infiltrates or focal retino-choroiditis have been reported as the commonest posterior segment finding in ocular CSD [9, 22]. – [23]. This was present in more than half (51.5%) of eyes with neuroretinitis in our series. Variable other terminology has also been used to describe these lesions including foci of yellow-white retinitis lesions and or choroidal infiltrates [5, 13] However, the incidence in eyes with CSD neuroretinitis has not been clearly established. We noted a significant presence of retinal infiltrates or focal retino-choroiditis at baseline in two-thirds of eyes that presented within one week from onset of symptoms and absence of these lesions in eyes that presented after 2 weeks (*p* = 0.034) indicating the early and transient nature of these lesions.

### Optical coherence tomography (OCT) and fluorescein angiography findings

OCT features in CSD Neuroretinitis are limited to case reports and small series. In our study, hyperreflective dots in the vitreous were seen in 50% of eyes at baseline. Similar findings were reported in a case of rapidly progressing CSD Neuroretinitis with clinically apparent vitritis at presentation [2]. In contrary, vitreous involvement on OCT preceded clinical visualization of vitritis or vitreous cells in 1 in 5 eyes from our series.

Presence of subretinal fluid in CSD NR is an established finding on OCT [27]. The percentage of eyes with baseline peripapillary SRF slightly exceeded those with subfoveal SRF in our series. This can be explained by the pathophysiology of fluid formation from leakage of vessels at or around the optic disc. N Kevin et al. in 2000 had also reported optic disc edema with peripapillary serous detachment as an early sign of systemic *Bartonella henselae* infection. ^28^

Baseline macula intraretinal fluid was less common and was present in eyes with co-existing peripapillary and subfoveal SRF. This may be related to disease duration and severity. An abnormal foveal contour was documented in more than two thirds of eyes. Z Habot et al. reported flattening of the foveal contour in all 8 eyes while subretinal fluid was seen in foveal region in 7 out of 8 eyes. ^27^

Significant reduction in mean CST was seen at 6 weeks (*p* < 0.001). The number of eyes with reduction of CST below 250 μm doubled from 5 to 10 eyes sighting foveal atrophy as a sequalae following resolution of subretinal fluid.

Leakage from the optic disc and retinal vasculitis were the predominant features seen on fluorescein angiography in this series. Similar findings were reported in a review by I Ksiaa et al. with early papillary and peripapillary telangiectasia with marked late leakage from optic disc and vessels in the absence of perifoveal leakage [8]. 

### Treatment

The efficacy of antibiotics and use of corticosteroids for the treatment of CSD Neuroretinits is still being debated. Majority of cases involving immunocompetent patients are found to be self-limiting and usually improve within two months [19]. Studies have found no significant difference in visual outcomes in treated vs. untreated patients with cat scratch optic neuropathy [17]. In contrast, from a multicentered retrospective study of 107 eyes in 86 patients with ocular Bartonellosis, Zohar et al. found that a combination of systemic antibiotics and corticosteroids in eyes with moderate to severe visual loss resulted in significant improvement of final vision compared to antibiotic monotherapy. Choice of antibiotics in her series included Doxycycline in 62% and additional rifampicin in 24%. Other agents included fluoroquinolones or macrolides monotherapy or in combination with rifampicin [3]. Chai LT reported that 17 out of 19 patients with ocular Bartonellosis received antibiotic treatment and Azithromycin was prescribed in 42%. Other agents used included doxycycline, ciprofloxacin, ceftazidime and cotrimoxazole. ^26^ Systemic corticosteroid therapy was used in approximately 58% which was higher than in our series. In our series, antibiotics were prescribed to all patients and Doxycycline was the preferred drug of choice. Addition of systemic steroids were used in around 4 out of 10 cases.

### Limitations

The small sample size and retrospective nature of the study with missing data was the main limitation. Multiple variable analysis was not possible and hence further prospective studies with larger samples size are required.

OCT images across retinal infiltrates or focal retino-choroiditis were not available for many cases and hence could not be analyzed in this series. Optical coherence tomography angiography (OCTA) images were also not available at the time of the study. Future studies should investigate the OCT and OCTA imaging characteristics of the retinal infiltrates or focal retino-choroiditis.

## Conclusion

Neuroretinitis secondary to cat scratch disease can occur in patients without prior history of contact with cats. Presenting visual acuity was moderate to poor with good final visual outcomes. Features of coexisting severe anterior uveitis were uncommon in adults and if present may prompt other etiological diagnosis. The absence of macular exudates at baseline appeared to be associated with peripapillary or subfoveal SRF in some eyes. Retinal infiltrates or focal retino-choroiditis were common, observed early and seemed to be transient. In some eyes, hyperreflective dots in the vitreous on OCT seemed to precede clinical visualization of vitreous involvement. Significant reduction in CST on OCT was seen at six weeks.

## Data Availability

The datasets used and/or analyzed during the current study are available from the corresponding author on reasonable request.

## Abbreviations

CSD: Cat scratch disease
NR: Neuroretinitis
VA: Visual acuity
CST: Central subfoveal thickness
OCT: Optical coherence tomography
OCTA: Optical coherence tomography angiography
RAPD: Relative afferent pupillary defect
OD: Optic disc
SRF: Subretinal fluid
IRF: Intraretinal fluid
ESR: Erythrocyte sedimentation rate
RNFL: Retinal nerve fiber layer
ONL: Outer nuclear layer
EL: Ellipsoid layer
